# Ensemble fuzzy multilayer neural perceptron with optimized feature selection for cardiac disease prediction using MRI and ECG data

**DOI:** 10.3389/fphys.2026.1719922

**Published:** 2026-04-22

**Authors:** J. K. Kiruthika, P. Thangaraj

**Affiliations:** 1 Department of Computer Science and Engineering, KPR Institute of Engineering and Technology, Coimbatore, Tamil Nadu, India; 2 Kangeyam Institute of Technology, Kangeyam, Tamil Nadu, India

**Keywords:** cardiac disease prediction, ensemble-based fuzzy neural network, MRI image analysis, median box filter, multilayer neural perceptron, grey segmentation, feature selection, optimal feature weighting

## Abstract

One of the major causes of death in the general population is cardiovascular disease. Life-threatening cardiac disease is influenced by several factors, including age, gender, blood sugar, cholesterol, heart rate, and more. There are so many factors that it can be challenging for specialists to assess each one. The current approach utilizes electrocardiogram (ECG) data and magnetic resonance imaging (MRI) image features but suffers from poor performance and high error rates. To address this problem, we employ an ensemble-based fuzzy multilayer neural perceptron (EFMLNP) model to predict cardiac disease. Initially, an image from the University of California, Irvine (UCI) Machine Learning Repository was selected to analyze the prognosis of cardiovascular disease. To effectively replicate the raw data values in the dataset, a median box filter (MBF) is used to pre-process the MRI dataset, reducing irrelevant values. The second stage, segmentation, uses adaptive mean gray segmentation (AMGS) to initialize two clusters for regions of interest and non-interest. The dataset is then tested using a feature-selection method based on recursive spectral spider optimization (RSSO) to identify the most pertinent characteristics for diagnosing heart disease (optimal reduced-feature splitting). Lastly, we examine a machine learning feature-extraction model and perform test analysis on the reduced features. The proposed EFMLNP method is evaluated using metrics including precision, recall, and receiver operating characteristic (ROC). The experimental outcome demonstrates that the accuracy is 98.3%, the precision is 97.15%, the recall is 98.43%, the F1-score is 96.34%, and the ROC is 0.96.

## 1 Introduction

Cardiovascular disease (CVD) is currently among the most common causes of death across the globe, and to enhance clinical outcomes, the detection and forecasting of risks should be conducted at an early stage. Innovations in medical imaging and physiological sensing have enabled the collection of complementary diagnostic information, including cardiac magnetic resonance imaging (MRI) and electrocardiogram (ECG) signals. MRI of the heart gives detailed structural and anatomic data, and ECG signals record electrical activity and functional cardiac behavior. The fusion of such heterogeneous modalities can potentially increase the accuracy of a diagnosis compared to single-modality prediction systems.

Recent years have seen machine learning and deep learning methods being extensively used on cardiac disease prediction based on imaging and physiological data. Convolutional neural networks (CNNs), recurrent neural networks (RNNs), and hybrid models have been shown to perform promisingly on the task of medical classification. Nonetheless, most available methods are based on a single data modality and are commonly exposed to high-dimensional feature representations, redundancy, and sensitivity to noise in physiological signals. These issues may have a negative impact on the generalization of models and the reliability of predictions.

Multimodal learning has become one of the promising strategies for incorporating complementary sources of medical information. However, multimodal fusion is not an easy task. It involves a thorough representation of features, identification of discriminative features, and solid classification systems that can cope with uncertainty in the medical data. Specifically, the overlapping features in merged datasets may amplify the computation cost and decrease the performance of classification, which emphasizes the necessity of optimizing the feature-selection strategies.

To overcome these issues, the current study suggests a multimodal cardiac disease prediction framework that combines the MRI image characteristics with ECG signal descriptors, using both an optimization-based feature-selection approach and a fuzzy ensemble neural classifier. Recursive spectral spider optimization (RSSO) is used to identify discriminative features in the fused feature space, and an ensemble fuzzy multilayer neural perceptron (EFMLNP) is the classification model. The framework suggested will enhance the strength of prediction by fusing structural imaging data and traits of physiological signals.

Validation on experimental data is done on publicly available cardiac MRI and ECG datasets with controlled training and testing conditions. Cross-validation and ablation studies are carried out to confirm whether multimodal feature fusion and RSSO-based feature selection help in improving and enhancing the performance of classification.

**FIGURE 1:**
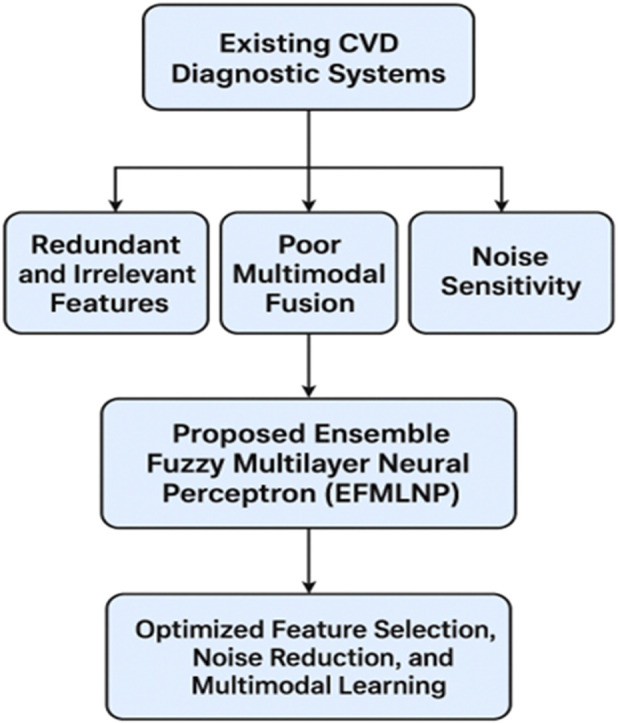
Problem identification and solution framework.

Figure 1 illustrates the problem identification and solution framework in CVD diagnosis. The most suitable attributes for each classification algorithm are selected through classifier-subset estimations using feature-extraction techniques. To assess classification performance, the caliber of the generated classification subgroup is considered. The procedure was carried out after comparing the results of these classifiers in categorizing the previously described heart disease dataset.

Transfer learning is the process of transferring data from one form to another to learn features that enable disease prediction. By applying feature fusion with machine learning and transfer learning, the performance of CVD detection can be improved. By considering all these factors, an efficient EFMLNP diagnosis model is presented, which trains the network with multiple intermediate layers. The neurons are designed to measure the impact value for a given feature, taking into account fuzzy values generated across various features and classes. Toward preprocessing, an efficient MBF algorithm is presented in this article. The method would read the pixels in the spectrum, analyze their distribution, and adjust them to improve image quality. Similarly, the technique would extract multiple features from both MRI and ECG data to generate the ensemble. The detailed workings of the proposed model are discussed in the remainder of the article. The most important contributions and novelties of this research are the following:

- A multimodal cardiac disease prediction framework, which combines structural MRI characteristics and physiological ECG signal characteristics, should be developed.
- Recursive spectral spider optimization (RSSO)-based feature-selection algorithm design is used to remove redundant features in the multimodal feature space.
- An ensemble fuzzy multilayer neural perceptron (EFMLNP) classifier is applied to enhance robust prediction during uncertain conditions of medical data.
- MRI image-based description is fused with ECG-based statistical features to increase cardiac disease classification at the feature level.
- Full experimental validation is conducted by comparing baselines, ablation, and K-fold cross-validation as a measure of the stability and effectiveness of the model.

## 2 Related work

### 2.1 Unimodal signal-based ECG and physiological models

A machine learning CVD risk factor detection model, CardioNet, is presented by Panwar et al. (2020) and utilizes a convolutional neural network to learn features for classification. A cross-domain transfer learning model is presented by Tadesse et al. (2019) toward diagnosing CVD. The method utilizes a convolutional neural network (CNN) model to learn features from ECG signals and train spectrograms. A phonocardiogram-based machine learning model that uses heart sounds to detect CVD is presented by Netto and Abraham (2021). A machine learning model-based prediction scheme, which considers smoking, alcohol, and other features by applying logistic regression, naive Bayes, a machine learning model, and random forest to perform classification, is given by Reddy et al. (2022).

A smartphone-based machine learning model is presented by Subasi et al. (2022), toward CVD diagnosis. A multi-input machine learning model is presented by Apostolopoulos et al. (2021), which uses multiple features to perform classification with random forests and neural networks. A detailed comparative study of CVD diagnosis is presented by Swathy and Saruladha (2022). A machine learning model that uses ECG characteristics for classification is presented by Dhiyanesh et al. (2024). A gene-association-based machine learning model that uses RNA sequences to predict disease is presented by Venkat et al. (2023). A machine learning model is presented by Ma et al. (2022), which uses a fundus image for classification with a CNN. An efficient machine learning model that uses different features is presented by Wong et al. (2020).

A DNFN-based classification model (Srilakshmi et al., 2022) selects and classifies features using the FC-HOA. A detailed review of CVD prediction and the available techniques is discussed by Dhiyanesh et al. (2025) and Denysyuk et al. (2023). An empirical mode decomposition-based machine learning model is presented by Li et al. (2023), toward CVD. The method considers multi-periodic signals and classifies abnormal heartbeats using a CNN. A multi-class classification model that uses multistage feature selection via fusion is presented by Hassan et al. (2022).

A machine learning ensemble-based CVD model, CraftNet, is presented by Li et al. (2020), which extracts handcrafted features and organizes them into a graph. Based on the graph, the method classifies the handcrafted features for CVD diagnosis. An empirical mode decomposition (EMD)-based CNN model is presented for CVD diagnosis by Abishek et al. (2023), which decomposes ECG signals using EMD and classifies them using intrinsic mode functions (IMFs). A time-series machine learning model using an LSTM is presented by Gandin et al. (2021) for CVD classification.

A machine learning-based segmentation model is presented by Dhamodharan and Goel (2022), who apply a machine learning model in the diagnosis of CVD. Similarly, a deep CNN (DCNN) has been used to detect CVD by Pawar et al. (2022). A watershed-based machine learning model is presented by Bensenane et al. (2022) for detecting cardiac stroke in the left ventricle using graph data. An LSTM-based decision support system, which extracts ECG features and trains an LSTM model using a neural network for classification, is utilized by Naveenkumar et al. (2022).

A machine learning feature fusion-based CVD detection method, which extracts various features from the Kaggle cardiovascular disease dataset and applies machine learning convolution filters (CVF) to perform CVD detection, is presented by Subasi et al. (2022). An automated machine learning model is presented by Karthik et al. (2022), which uses a deep belief network with the Improved Swallow Swarm Optimization (ISSO) algorithm for classification.

Table 1 presents a comparative study of various machine learning (ML) and deep learning (DL) models for cardiovascular disease (CVD) classification. Each reference is assessed based on classification accuracy, time complexity, training and testing strategies, and key drawbacks. Table 1 highlights how different architectures, such as CNNs, RNNs, LSTMs, GANs, and transfer learning methods perform under varying data conditions (image, sensor, and multimodal inputs), emphasizing common limitations such as data dependency, noise sensitivity, and interpretability issues.

**TABLE 1:** Comparative analysis of CVD classification models.

Reference number	Model used	Analysis of CVD accuracy classification	Time complexity	Analysis of training and testing model	Drawback
Tadesse et al. (2019)	Transfer learning with CNN	Accuracy improved with transfer learning	High	Cross-domain validation approach	Requires a large labeled dataset and domain shift issues
Netto and Abraham (2021)	CNN and RNN	Accuracy varies with noise levels	High	Training on clean data and testing on noisy data	High false result
Apostolopoulos et al. (2021)	CNN and MLP	High accuracy with integrated data	High	Multimodal data training and testing	Integration challenges of heterogeneous data sources
Ma et al. (2022)	CNN for image analysis	High accuracy in risk assessment	High	Training on fundus images and testing on a diverse population	Variability in image quality and demographic differences
Wong et al. (2020)	CNN and GANs	Accuracy varies with dataset size	High	Training on image datasets and testing on unseen images	Limited labeled image datasets and model interpretability issues
Subasi et al. (2022)	CNN and LSTM	Accuracy is influenced by sensor quality	Low	Training on smartphone data and testing on real-world scenarios	Dependency on smartphone sensors and data quality issues
Panwar et al. (2020)	CNN and deep neural network (DNN)	High accuracy, but overfitting was observed	Moderate	Training on limited data and testing on unseen data	Limited dataset and overfitting risk
Reddy et al. (2022)	Support vector machine (SVM), CNN, and DNN	Accuracy metrics not specified	Moderate	Comparative study with multiple models	Limited feature selection and potential bias in the dataset
Dhamodharan and Goel (2022)	CNN for ultrasonic image analysis	Accuracy depends on image quality	Moderate	Training on ultrasonic images and testing on diverse data	Limited dataset and variability in image quality

Table 2 summarizes the performance and characteristics of various DL and ML models for cardiovascular disease (CVD) classification. It presents each model’s accuracy, computational complexity, training and testing strategies, and key limitations. Table 2 highlights the impact of advanced approaches such as identity pre-training, ensemble methods, joint segmentation, and feature fusion on predictive performance, while also emphasizing challenges, including dataset limitations, computational requirements, noise sensitivity, and interpretability issues.

**TABLE 2:** Relative assessment of DL and ML models for CVD classification.

Title	Model used	Analysis of CVD accuracy classification	Time complexity	Analysis of training and testing model	Drawback
Dhiyanesh et al. (2024)	Deep learning model with identity pre-training	High accuracy with identity-based features	High	Training with identity pre-training, testing on ECG data	Requires a large, labeled ECG dataset and computationally intensive
Srilakshmi et al. (2022)	Hybrid DL model with joint segmentation	High accuracy with joint segmentation	High	Training with joint segmentation and testing on clinical data	It does not support a large dataset.
Li et al. (2020)	Ensemble DL model	High accuracy with the ensemble approach	High	Training with an ensemble model and testing on diverse data	Requires a large dataset and potential for overfitting
Gandin et al. (2021)	Time-series DL models	Accuracy varies with model interpretability	High	Training on ICU data and testing on unseen patients	Interpretability challenges and data privacy concerns
Pawar et al. (2022)	Deep learning with the watershed algorithm	High accuracy in stroke volume calculation	High	Training with MRI data and testing on clinical data	Did not improve image quality
Venkat et al. (2023)	ML models with genetic data integration	Accuracy depends on genetic data quality	Moderate	Training on genetic data and testing on clinical data	Limited genetic data availability and ethical concerns
Li et al. (2023)	Deep learning with EMD features	Accuracy depends on EMD feature quality	Moderate	Training with EMD features and testing on ECG data	Accuracy result is lower
Hassan et al. (2022)	Feature selection with deep learning fusion	High accuracy in early detection	Moderate	Training with feature fusion and testing on clinical data	Did not solve class imbalance issues
Abishek et al. (2023)	Deep learning with modified ECG signals	Accuracy depends on signal modification	Moderate	Training with modified ECG signals and testing on ECG data	Lower precision and accuracy performance
Swathy and Saruladha (2022)	Various ML and DL models	Accuracy varies across models	Variable	Comparative analysis of multiple approaches	Variability in dataset quality and size
Dhiyanesh et al. (2025)	Review of various ML approaches	Accuracy varies across studies	Variable	Review of numerous ML approaches	Lack of primary data, variability in study methodologies
Denysyuk et al. (2023)	Review of ECG-based algorithms	Accuracy varies across algorithms	Variable	Review of ECG-based diagnostic algorithms	It does not support multimodal data

### 2.2 Multimodal and feature fusion-based approaches

Jabbar et al. (2025) proposed an automated cardiac disease prediction framework, termed GenDeep, that integrates an unsupervised generative adversarial network (GNN) with a DeepLab segmentation model for cine-MRI analysis. The approach enhances feature learning and segmentation accuracy for cardiac pathology classification; however, it relies solely on MRI data, without incorporating multimodal physiological information or optimizing features.

Zia et al. (2025) utilized EfficientNet with compound scaling in a digital twin-based cardiac arrest prediction framework. Their model effectively learns discriminative features from cardiovascular images while maintaining computational efficiency. However, it is limited to image-based analysis and does not address multimodal data fusion or feature selection.

Hadi et al. (2025) introduced a DenseNet-based deep learning model for cardiovascular disease detection using clinical data. The dense connectivity enables effective feature reuse and improved prediction performance; however, the approach does not exploit medical imaging modalities or advanced preprocessing and optimization techniques required for high-dimensional cardiac data.

Despite the various studies conducted to predict multimodal cardiac disease based on MRI and ECG data, most of the current methods are based on naive feature-level or early fusion schemes, which simply combine heterogeneous features across different modalities without considering modality-specific relevance and noise properties. These types of fusion are extremely susceptible to ECG noise, MRI artifacts, and inter-modality feature imbalance, with the net effect of occasionally introducing redundant or conflicting representations. Furthermore, most of the previous models do not have effective feature optimization methods, which results in high dimensionality and overfitting. Meanwhile, traditional neural networks cannot deal with the uncertainty and nonlinear interactions that multimodal cardiac data exhibit. In turn, such constraints greatly reduce classification accuracy and the performance of generalization in clinical conditions.

### 2.3 Limitations

- Accurate prediction of cardiovascular disease remains challenging due to the high dimensionality, heterogeneity, and noise present in clinical, ECG, and imaging datasets, which complicates model generalization.
- Many conventional machine learning approaches rely on manual feature engineering or single-modal data, which limits their ability to capture complementary diagnostic information and often results in reduced prediction robustness.
- Existing feature-selection methods, including clustering-based weighting strategies, may reduce feature dimensionality but often fail to preserve clinically significant multimodal relationships, leading to sub-optimal classification performance.
- Several prior studies report limited evaluation protocols and insufficient validation, which affects reproducibility and reliability in real-world clinical scenarios.

### 2.4 Summary and motivation of the proposed framework

Despite significant progress in cardiac disease prediction using MRI and ECG data independently, limited research has focused on efficient feature-level fusion of these modalities combined with optimized feature selection. High-dimensional multimodal feature spaces often introduce redundancy and noise, which negatively affect classification performance. Therefore, a lightweight multimodal framework integrating robust feature selection and ensemble learning is required to improve prediction reliability while maintaining computational efficiency.

## 3 Proposed method using EFMLNP

The proposed system for predicting cardiovascular disease uses ensemble fuzzy multilayer neural sensitivity. To predict heart disease with high accuracy, the neural network in this system is trained on a robust set of clinical data using backpropagation. The proposed model reads the provided MRI image and ECG data. The method applies the RSSO analysis algorithm to the input to improve the quality of image features, and MBFs removes the noise from the image. Furthermore, the process uses the adaptive gray segmentation algorithm to group image features. The features of deposits at different locations are extracted from segmented images. Similarly, the method extracts pressure and heart rate data from the ECG. Using the extracted features, the process generates ensembles as a single feature vector. Generated ensembles are used to train the DNN, and the neurons are designed to analyze the cluster-based enhanced feature weighting mechanism (CEFWM). During the test phase, the neurons compute the coefficient of evidence for class membership (CECIM) values for various CVD classes, generate fuzzy values for different features, and calculate the CECIM value accordingly.

**FIGURE 2:**
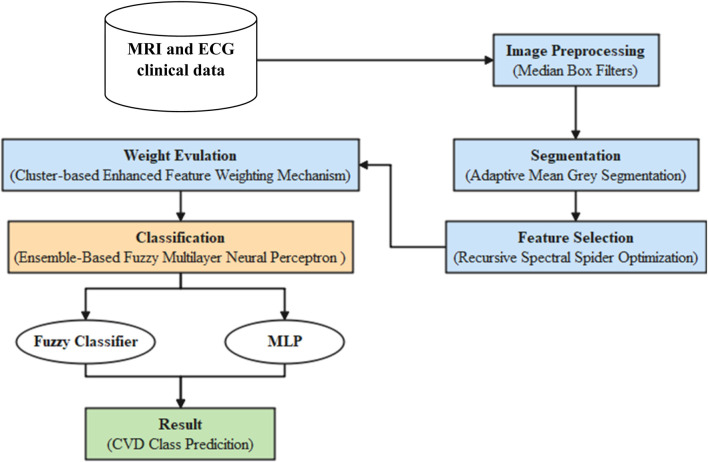
Proposed EFMLNP method-based architecture diagram.

The proposed cardiac disease prediction framework consists of six stages: preprocessing, segmentation, feature extraction from MRI and ECG signals, multimodal feature fusion, RSSO-based feature selection, and EFMLNP classification. Figure 2 illustrates the overall workflow of the proposed system. The process begins by collecting the dataset and proceeds to the preprocessing stage, in which MBF is used for noise reduction and image normalization. The second step is segmentation, based on AMGS, used to segment the regions of the original pre-processed images. The third step is feature selection, which is based on RSSO and is used to evaluate the weights and select the maximum feature weights based on the threshold values. Weight calculation is based on CEFWM. Before classification training, the SoftMax classifier’s values are used to estimate the weights. Finally, the classification step is based on an ensemble fuzzy multilayer neural perceptron combined with fuzzy and multilayer perceptron (MLP) methods topredict the disease and determine whether to select it.

### 3.1 Preprocessing median box filters

Median box filter (MBF) refers to an adaptive median filtering configuration designed for MRI noise suppression in the proposed pipeline. The MBF uses the median of its neighborhood pixels to estimate each pixel’s value. The average of the adjacent pixels inside the designated square size is then used to replace each original pixel. The MBF reduces noise and blur by weighting averages across individual pixels. The mean value of the pixels within the neighborhood defined by the mask size (m columns, n rows) is then determined. It is crucial to divide the output value by the sum of its nearby values to normalize it. The values of adjacent pixels determine the median box, which is subsequently allocated to the relevant pixel in the filtered image. An MBF with a kernel size of 3 × 3 is applied, in which the median value within the neighborhood window replaces each pixel’s intensity.

Let ${\left[{\;L:C}_{ij}\right]}_{iIJ}$ be an image matrix (img), $C_{ij}$ is an unsigned integer, and if the values between $255\;\left(0\leq C_{ij}\leq 255\right)$ , then $C_{ij}$ is called a noise entry of C , and if $C_{ij}=0\text{ or}C_{ij}=255.$

Algorithm steps

Start
Step 1: Read the noise image matrix ${\left[{\;L:C}_{ij}\right]}_{iIJ}$ , where $\left\{i,j\right\}\geq 1$

Step 2: Binary matrix $B:{\left[{\;M}_{ij}\right]}_{i*J}\;of\;C$

If c is a noisy image, then the matrix $M:{\left[{\;M}_{ij}\right]}_{i*J}$ is a binary matrix of M

$M_{ij}=\left\{\begin{array}{c} 0,{\;C}_{ij}\;is\;a\;noise\;entry\;of\;C\\ 1,otherwise \end{array}\right.$

$u\left(i,j\right)=Mf\left(i,j\right)=MedianBox\left\{v\left(i-k,j-1\right),\left(k,i,j\right)\right\}$

$MB=\frac{1}{{MedianBox}_{Hight}*{MedianBox}_{width}}\left[\begin{array}{ccc} 1 & 1 & 1\\ 1 & 1 & 0\\ 1 & 0 & 1\; \end{array}\right]$

$\min_{v}{\left\|\nabla v\right\|}_{1}\left.+\right||M^{k}-v{\|}_{1}+\frac{\sigma }{2}\left\|\;\right.Rfc-M_{c}{\|}^{2}$

End if
Stop

The MBF is selected as the preprocessing module because cardiac MRI images often suffer from speckle noise, acquisition distortions, and pixel-level fluctuations, which degrade the clarity of anatomical boundaries. Unlike linear filters, which tend to blur edges, MBF preserves structural details while selectively removing impulse noise, making it highly suitable for medical images, where edge preservation is essential for subsequent segmentation. Its nonlinear median-based smoothing improves contrast uniformity, enhances the visibility of the myocardial regions, and reduces the computational burden for downstream segmentation and feature extraction. Therefore, MBF is an appropriate and reliable choice for ensuring noise-free, edge-preserved MRI images before further processing.

**FIGURE 3:**
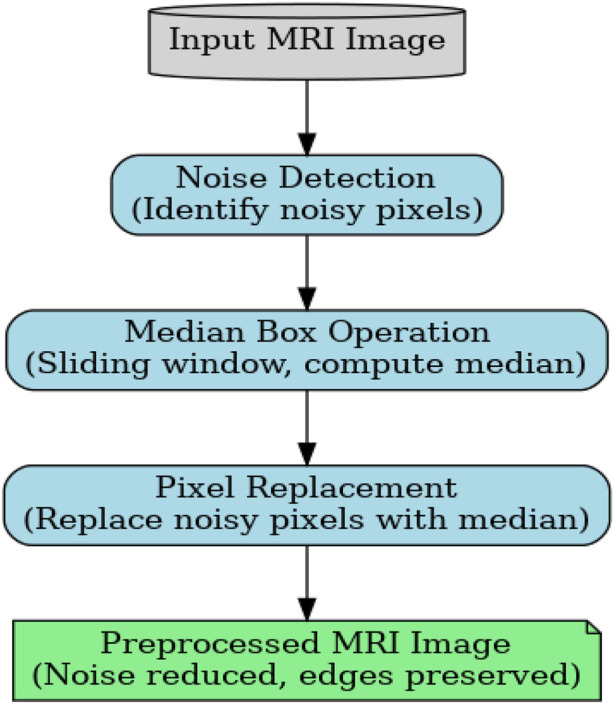
Need for preprocessing.

Let us assume *B* is the binary matrix, the row (*i*) and column (*j*) indices, directly corresponds to a pixel position, *v(i − k, j − 1)* is the neighboring pixels, MB is the mask normalization, ∇v is the gradient of a smooth image, $M^{k}$ is the result iteration, Rfc is the image component, and σ is the current image component. The result, M_ij_, obtained by applying the median filter M_c_ to the image at the *k*th iteration, reduces the additional noise introduced during the transformation process. The Median Box Filter (MBF) and guided filtering work together to fix areas of the image that are hidden or noisy. So, as shown in Figure 3, the steps in the process are as follows: breaking the image up into smaller areas or boxes for processing; and using a fast median filtering algorithm on each box to improve the quality of the image and filter out noise.

### 3.2 Adaptive mean gray segmentation

The pre-processed MRI image has been used to segment the features, supporting feature extraction. To perform this, the method initializes two clusters: one for interested regions and one for non-interested regions. The process generates the gray values from the image and the gray histogram. Using the histogram, the method identifies the minimum and maximum values within each histogram group based on the number of pixels in that group. Using the least maximum value, the process reads the pixels of each image, identifies the group of pixels, and indexes them into the cluster. The clustered and segmented image has been utilized for CVD detection.

Algorithm steps
Input: P_img_ denotes the pre-processed image.
Result: Segmented image Simg

Start
Read P_img_.
Hist = generate histogram (P_img_)
Identify least max value Lmv = $i=1if\;Hist\left(i\right).count>$ $20\;\&\&\;Hist\left(i\right).value>220i=size\left(Hist\right)$

Initialize Cluster set cs = {C1, C2}
For each pixel pi
If P_img_(pi). value < Lmv then

$C_{1}=C_{1}\cup \left\{p_{i}\right\}$

Else

$C2=\sum_{pi\in C2}\left(p_{i}+p_{i}\right)$

End if
End for
Stop

Here, Cluster C1 represents the region of interest (RoI), corresponding to cardiac structures such as the myocardium and ventricles, while Cluster C2 denotes non-relevant background regions.

**FIGURE 4:**
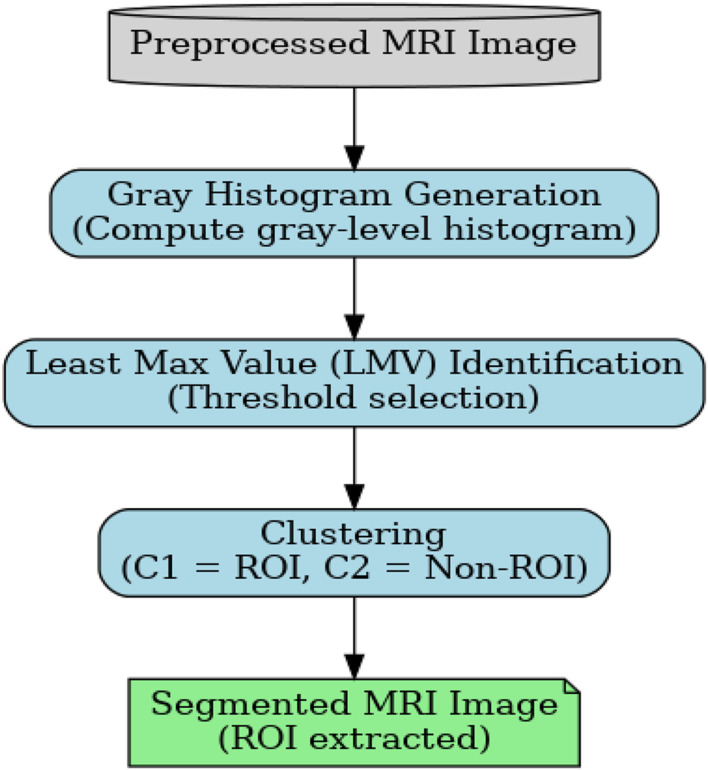
RoI extraction from segmentation.

The above-discussed algorithm and Figure 4 describe segmentation by computing the Least Max Value (LMV) from the gray histogram. Let H(g) denote the gray-level histogram of an image, where gϵ{0,1,…,L−1}. In is defined as the gray level corresponding to the minimum among the dominant local maxima of the histogram. The computed LMV is employed as a global threshold for image segmentation, enabling effective separation of regions in images with uneven intensity distributions. 

### 3.3 Recursive spectral spider optimization

The RSSO method is presented as a reproducible feature-selection strategy rather than a biologically descriptive model.

Spider-based behavior: The spider weights in the method are proportionate to the solution estimate, reflecting this tendency. Gender is a dividing line among spiders, and individual behaviors within the group are distinct. Individual evolution operators are used for males and females to create this distinction. On average, 70% of the population is female.

#### 3.3.1 Spiders’ recursive behavior

RSSO is a population-based metaheuristic that minimizes a biologically inspired supportive searching and communication behavior of social spiders. In RSSO, every individual candidate solution is assumed as a spider placed in a multidimensional feature space, with every dimension of this space connecting with a feature variable. The challenge of RSSO is finding an optimal set of features that maximizes a certain predefined function, which is labeled as a fitness function, while ensuring a minimum redundancy. The key features are normalized area of the deformation (NAD), average curvature of the deformation (ACAD), normalized volume deformation (NVD), and average curvature of volume deformation (ACVD). They are evaluated through RSSO, through which spiders communicate through a vibration across a virtual path. The vibration carries solution quality information, and spiders are able to adjust their location according to local and global optima.

Each individual is given a weight in relation to fitness, and tougher spiders with a higher fitness value have greater weights and thus a stronger impact on the entire population. For simulating the realistic behavior in the social context, the population is considered on the basis of gender and male/female spiders, each following different movement and interaction policies. Empirical studies related to the natural colonies of these spiders demonstrate that the females in the spider colony form the major part of the population and play a major role in the exploration process. Hence, in RSSO, it is assumed that 70% of the population is female and 30% is male.

A random sample of females, denoted ${No}_{fem}$ , is selected from 70% to 90% of the total population N . Consequently, ${No}_{fem}$ is determined using Equations 1–3 that follow:

The fitness values of the solution for each spider are determined by the weight assigned to them individually:

$${No}_{fem}=neighbor\left[0.9-random\;025\right]*N,$$ (1)

$$w_{x}=\frac{{good}_{x}-bad}{Best-bed},$$ (2)

$$S_{n}^{a+1}=\left\{\begin{array}{c} S_{n}^{a+1}+\alpha .f_{n,x}.\left(s_{n}-S_{n}^{a}\right)+\beta .v_{n,x}.\left(s_{n}-S_{n}^{a}\right)+\delta .\left(random-\frac{1}{2}\;possibleps\right),\\ S_{n}^{a}-\alpha .f_{n,x}.\left(s_{n}-S_{n}^{a}\right)+\beta .v_{n,x}.\left(s_{n}-S_{n}^{a}\right)+\delta .\left(random-\frac{1}{2}\;possible\;1-ps\right), \end{array}\right.$$ (3)

where ${No}_{fem}$ is a random subset of female spiders, α,β,δ,and rand are random numbers between M and F− vibration $f_{n,x}$ , the number of iterations, and the number of individuals, x is each spider, $w_{x}$ is the weight, ${fs}_{n}^{x}$ is the fitness score of the spider, n is the number of features, s is the spider, $\left(A\left({fs}_{n}^{x}\right)\right)$ is a function to evaluate the solution fitness, $v_{n,x}$ is the velocity component of the spider movement, and possibleps,possible 1−ps are the possible step variations in the position, which represent the closest and best spiders with more weight than the general web, respectively.

$${Best}_{x}=\max_{\left.w\left\{1,2,..\right.n\right\}}\left(A\left({fs}_{n}^{x}\right)\right),$$ (4)

$${worst}_{x}=\min_{\left.W\left\{1,2,..\right.n\right\}}\left(A\left({fs}_{n}^{x}\right)\right).$$ (5)

Equations 4, 5 compute the best $\left({Best}_{x}\right)$ and worst $\left({worst}_{x}\right)$ spiders based on fitness score. The weighted dataset is filtered using feature weights (*w*) and the number of values (*n*) to determine the most and least significant features.

#### 3.3.2 Feature weight calculation

This section presents the method for determining feature weights. According to RSSO logic, different features in the dataset have varying importance for predicting heart disease. A 0 to 1 weight is assigned to each characteristic. Features closer to 1 are of greater importance. Weights that are near 0 have minimal impact on heart disease prediction.

The individual feature weights are determined using Equation 6. Consider the set of features F.F = $\left\{n0,n1,n2...ni\right\}\;and\;\left(n\;>\;0\right).$

Nf wei=a∑no,n∈faa0+a1+…+ax. (6)

Let (Ma ∪ Fe) be the total number of records, and let A be the number of each feature in the dataset, as calculated in Equations 7, 8.

$${fw}_{\left(val=x\right)}=\frac{X}{\text{Ma}\cup \text{Fe}},$$ (7)

$$w\;\left(tx\right)=wei\;\left(a\right)*wei\;\left(val\right).$$ (8)

Let us assume ni is an individual feature, a is the current feature, $a_{0}+a_{1}+\ldots +a_{x}$ are the contributions of all features, f is the features, Nf is the number of times the feature value, feature weights $w\left(tx\right)$ , and feature value weights $w\left(val\right)$ give the total feature weights $\left(W\left(n\right)\right)$ .

#### 3.3.3 RSSO performance

At every step, RSSO recursively selects features and then trains SVMs on the remaining features to re-rank them. The RSSO will not modify a weak feature; it is responsible for a lack of functionality. Every spider will be assigned a weight W, which is computed from the fitness value, fi, of the solution it represents. Higher fitness means larger weights, and hence larger influence in the network. The computation of weight ensures that the spiders of better feature subsets lead the search process compared to the inferior solutions. The recursive mechanism for updating spider positions is carried out frequently by the RSSO algorithm in multiple iterations. During each iteration, spiders will perceive the vibrations generated by other spiders and adjust their position. Some of them will be eliminated through recursive evaluation and ranking of features according to their contribution to classification performance.

Algorithm steps

Start

R:=Ranked feature=∅

$Fr-Remaining\;Feature=\left\{1,2,3,4\right\}$

S:=the score of a criterion function for a particular feature set

Count of remaining features, f:=n

Train the overall count score $C_{s}=C\left\{C_{x}\left(S\right),y_{s}\right\}$

Compute the weight and rank the features based on the values $\delta =\left\{f_{1},f_{2},..{.,f}_{n}\right\}$

Set f=f−1 and a=1.

Let f_1_ = f∖{f_i_}, where f_i_ is the feature being removed from f.

$C_{2}=C\left\{C_{x}\left(S\right),{\;y}_{s}\right\}$

If $C_{1}<C_{2}$

$A=A\cup f_{a}$

Else if

$A=A\cup f_{a},C=C-f_{1}$

Else

C=C+1

End if
End
Stop

The SVM classifier is employed solely as a fitness evaluator within the RSSO framework to assess candidate feature subsets. After eliminating the likelihood of weak features, their significance is assessed by comparing their values to determine which are most important. The procedure is repeated until a feature is identified that, in its absence, will not negatively impact classification performance. Given that characteristics are initially sorted according to their values of $wei\left(a\right)$ , Let’s assume $C_{s}$ is the count score, f is the features, and A is the selected feature subset. The key MRI features extracted are: NAD, ACAD, NVD, and ACVD. These features describe the structural and shape-based characteristics of the heart. Additionally, ECG features, including heart rate (HR) and surrogate blood pressure (BP), are used as auxiliary functional indicators. The BP values are estimated using correlations from ECG waveforms. These features are used as inputs to the RSSO feature selection and subsequent EFMLNP classification pipeline.

### 3.4 Ensemble-based fuzzy multilayer neural perceptron (EFMLNP)

The proposed ensemble-based fuzzy multilayer neural perceptron (EFMLNP) model combines fuzzy feature representation with an ensemble multilayer perceptron (MLP) classifier to improve the robustness of cardiovascular disease prediction. The optimized feature subset obtained from the RSSO feature-selection stage is used as the input to the EFMLNP model.

The EFMLNP architecture consists of five layers: an input layer, a fuzzy membership layer, two hidden layers, and an output layer. The input layer receives the selected multimodal feature vector derived from MRI and ECG signals. The fuzzy membership layer transforms numerical feature values into membership degrees in the range [0,1], representing the uncertainty associated with clinical and imaging features. This transformation enables the neural network to handle variability and noise in medical data more effectively.

Algorithm steps

Start

Read RSSO, Teg, E.
P_img_ = Apply MBFs (Teg)
Simg = AMGS $\left(Pimg\right)$

[NAD, ACAD, NVD, ACVD, Bp, Heart Rate] = Feature Extraction $\left(Simg\right)$

For each class c
For each feature f
Compute fuzzy value Fzv = $\frac{\begin{array}{c} Size\left(EnsembleSet\right)\\ EnsembleSet\left(i\left(f\right)\right).value\\ i=1 \end{array}}{Size\left(set\right)}$

End for
For each neuron n

Compute artery CVD impact measure (ACIM) = $\frac{\text{Dist}\left(\text{Fzv}\left(\text{NAD}\right),E\left(\text{NAD}\right)\right)}{\text{Dist}\left(\text{Fzv}\left(\text{ACAD}\right),E\left(\text{ACAD}\right)\right)}\times 100$

Compute valve CVD impact measure (VCIM) = $\frac{\text{Dist}\left(\text{Fzv}\left(\text{NVD}\right),E\left(\text{NVD}\right)\right)}{\text{Dist}\left(\text{Fzv}\left(\text{ACVD}\right),E\left(\text{ACVD}\right)\right)}\times 100$

Compute ECIM = $\frac{\text{Dist}\left(\text{Fzv}\left(\text{Bp}\right),E\left(\text{Bp}\right)\right)}{\text{Dist}\left(\text{Fzv}\left(\text{HR}\right),E\left(\text{HR}\right)\right)}\times 100$

Compute CECIM = $\frac{VCIM}{ACIM}\times ECIM$

End for each
End for each
Class C = Choose the class with the maximum CECIM.
Stop

The CVD detection model estimates the CECIM measure for various CVD classes based on the features considered to classify images. Let’s assume E is the ensemble set, Pimg is the pre-processed image, Simg is the segmented image, n is the number of neurons in intermediate layers, Fzv is the fuzzy value of the feature, and C is the predicted CVD class.

The degree of shared information between features and the class-based rule is measured by the fuzzy decision-making relevancy connection set of If … Then. The rule presented in Equation 9:

$$R_{x}:IF\;\left(a∼f_{a}\right)\;THEN\;\left(b_{n}=T_{n}\left[1,\frac{1}{\sqrt{M_{x}}}x^{t}f_{n}\right]\right).$$ (9)

Let us assume $f_{a}$ is the feature value, $b_{n}$ is the output label, $M_{x}$ is the number of features, $T_{n}$ is the transformation function, and $f_{n}$ is the function.

These characteristics are deemed unnecessary and do not require selection. The redundancy relation measures the quantity of common information between features. Using a fuzzy set, the dependency relation calculates the degree of class membership using the Equations 10–12 for the feature subset. Let’s assume $w_{x}$ is the feature weight, $\bar{M_{xy}}{\left.+\bar{M_{xy}}\right)}^{2}$ is the mean feature value, $x_{f}$ is the feature-specific value, and m is the mean.

$$F=\sum_{X=1}^{m}\left(\max\left(w_{x}\bar{M_{xy}}{\left.+\bar{M_{xy}}\right)}^{2}\right.\right),$$ (10)

$$F1\sum_{X=1}^{m}\left(\min\left(w_{x}\bar{M_{xy}}{\left.-\bar{M_{xy}}\right)}^{2}\right.\right),$$ (11)

$$FD=\frac{F}{F1+F},$$ (12)

$$F=\left(\frac{x_{f}-\sup\left(M_{xy}\right)}{\sup\left(M_{x}\right)-\inf\left(M_{y}\right)}\right),M=\sup\;\left(w_{x}\left(\frac{x_{f}-\sup\left(M_{xy}\right)}{\Delta \left(M_{y}\right)}\right)\right).$$ (13)

An ensemble-based approach derived from machine learning principles is applied, integrating multiple moderately performing ranking sets from individual multilayer perceptrons (MLPs) to form a more robust ranking model. The fuzzy membership–ranking relationship calculated using Equation 13 is considered under the following conditions.

- Membership value = 0.9 with a ranking position of 1,
- Membership value = 0.1 with a ranking position of 6, and
- Membership value = 0 with a ranking position of 13.

Here, $\bar{R}$ (x) calculated using Equation 15 denotes the normalized ranking feature, and Fl calculate using Equation 15 represents the fuzzy inference layer.

$$Fuzzy\;Layer\;\left(Fl\right)=\left\{\begin{array}{c} 1-{\left[1+s{\left(a-b\right)}^{2}\right]}^{-1},1\leq a\leq 0,\\ 1-c|na+d,Otherwise, \end{array}\right.$$ (14)

$$\bar{R}\;\left(x\right)=Rank\;\left(\sum_{f}^{f=J}fuzzy\;\left(S_{xy}\right),\sum_{x}^{x=1}\sum_{y}^{y=1}fuzzy\;\left(S_{xy}\right)\right).$$ (15)

A fuzzy change is used to combine all of the fuzzy multilayer perceptron (FMLP) ranks into the final rating. Ranking computation: $S_{xy}$ denotes the *i*th alternative and *y*th ranking, while rank (R, S) denotes A’s position in set S.

Its layer depth defines each enhanced fuzzy multilayer perceptron (EFMLP) and the neuron count within each layer. Accordingly, the three EFMLP models developed for this ensemble differ in their input dimensions: 128, 144, and 156, and in the configuration of their hidden neurons. Another distinction among these models lies in the batch sizes used during mini-batch gradient descent: MLP 1 uses a batch size of 128, MLP 2 uses 64, and MLP 3 again uses 128. For each sample, each model assigns a label; the ensemble then compares these labels to reach a final decision. The model is calculated to find the value that yielded the most updated prediction based on the largest number of quantities.

This is accomplished by reducing the hyperplane’s inaccuracy when applied to the training dataset.

$$\in \left(w\right)=-\sum_{n}^{c}x\in wt_{x}w^{t}x_{j}.$$ (16)

If $\in \left(w\right)$ = 0, this indicates that the classes are totally divided by the hyperplane. Usually, this minimizing process is done iteratively, moving closer to the minimum with each iteration. Herein, whereas the Equation 16 shows ∈ (*w*) which is the hyperplane for the weight vector, w is the weight vector, c is the total number of classes, and $t_{x}w^{t}x_{j}$ is the true class label.

$$\Delta w=\eta \;\left({true}_{n}-{pred}_{n}\right)w^{t}x_{j}.$$ (17)

The Δw has been calculated using the Equation 17 where as, Let η represent the learning rate, where true_j_ denotes the actual class label, and pred_j_ indicates the predicted label. Define a universal set U = {x_1_, x_2_, x_3_, … , x_n_}. A fuzzy set A ⊆ U can be expressed as a collection of ordered pairs {(xᵢ, μA (xᵢ))}, with xᵢ ∈ U and a membership function μA: U → [0,1]. The value of μA (x) quantifies the extent to which element x belongs to the fuzzy set A.

A Type-II fuzzy set, denoted as A′, extends this notion and is formulated as in the Equation 18:

$$A^{\prime }=\left\{\left(x,\mu \right),\mu A^{\prime }\left(x,\mu \right)|x\in U,\mu \right\}\text{subject to the condition }0\leq \mu A^{\prime }\left(x,\mu \right)\leq 1.$$ (18)

**FIGURE 5:**
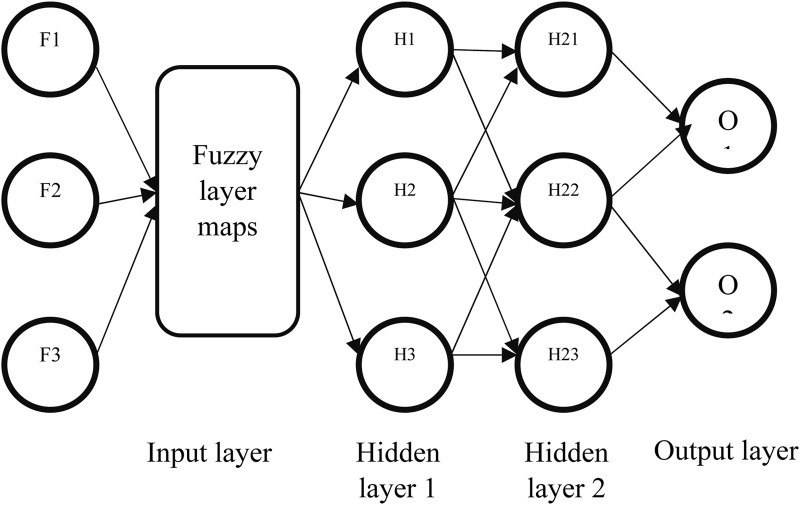
Fuzzy MLP structure.

In essence, creating a type-II fuzzy set involves constructing a type-I fuzzy set and assigning each element an interval of membership values, defined by a lower and an upper membership degree, rather than a single crisp value. In this study, the multilayer perceptron learns by considering each input sample’s membership degree in the disease classes (e.g., disease vs. no disease). Additionally, during the weight update process, gradient descent benefits from incorporating membership values. Specifically, features with ambiguous membership, typically approximately 0.5, exert a reduced influence on learning. This helps the model focus more on features with higher certainty. As illustrated in Figure 5, the fuzzy MLP assigns membership degrees by applying fuzzy clustering at each layer of the neural network, except for the output layer. Table 3 summarizes the proposed workflow.

**TABLE 3:** Summary of proposed work.

Stage	Algorithm	Key function
Preprocessing	Median box filter (MBF)	Reduces noise and smooths edges
Segmentation	Adaptive mean gray segmentation (AMGS)	Gray histogram + LMV threshold and RoI detection
Feature optimization	RSSO (Spider behavior, feature weights, and performance)	Recursive feature selection, weight assignment, and SVM retraining
Classification	EFMLNP (fuzzy + MLP)	Learns from optimized features and ensemble decision making
Results	Evaluation metrics	Accuracy, recall, precision, F1, and ROC

The EFMLNP is selected as the classification module due to its ability to model nonlinear relationships and handle uncertainty within medical data. Cardiac MRI features often exhibit fuzzy boundaries, overlapping intensities, and nonlinear class separations, which make traditional classifiers unstable. By integrating fuzzy membership functions within an ensemble MLP architecture, EFMLNP enhances interpretability, improves decision reliability, and reduces classification variance. The ensemble strategy improves generalization by combining multiple weak learners, while the fuzzy layer helps manage the ambiguity inherent in cardiac structural variations. These combined strengths allow EFMLNP to outperform conventional single-model classifiers, making it a highly suitable choice for robust cardiac disease prediction.

## 4 Results and discussion

The proposed EFMLNP models for CVD diagnosis have been analyzed for performance across different sample sizes within various classes. A detailed analysis is presented in this section. The proposed method can be compared with previous methods, such as the DNFN, LSTM, CNN-LSTM, and DCNN, to evaluate its performance.

### 4.1 Dataset description

In this research, cardiac disease prediction is formulated as a multi-class classification task, using cardiac MRI images as the primary modality, with ECG signals incorporated as auxiliary physiological features and not used as disease labels. Because patient-level paired MRI–ECG datasets are limited in public repositories, this study evaluates multimodal feature learning using independently sourced MRI and ECG datasets. ECG features are used as auxiliary physiological descriptors to test the robustness of multimodal feature fusion rather than patient-specific fusion

**FIGURE 6:**
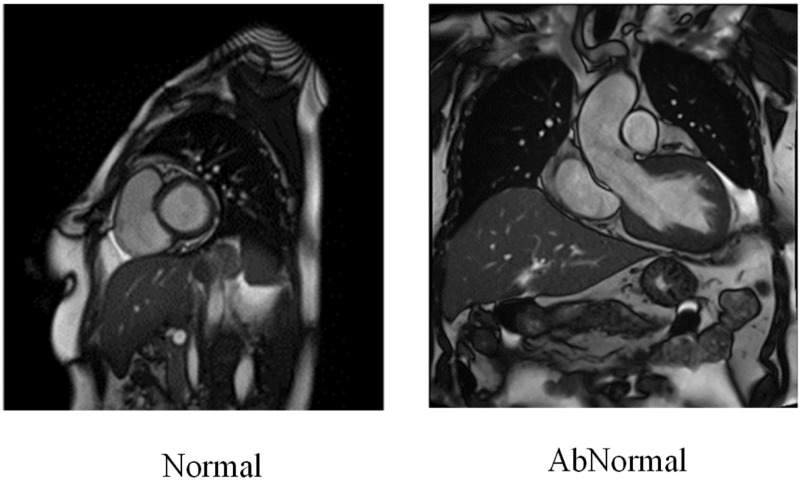
MRI image dataset.

The experimental assessment employs a multimodal cardiac data set comprising cardiac MRI images and ECG signals. The MRI images in the fastMRI dataset, comprising 7,500 images of normal and abnormal MRI scans, as shown in Figure 6, can serve as a basis for a comparative study or multimodal research. The CVD detection provides a detailed set of cardiac MRI images, with an emphasis on cardiac motion tracking and segmentation, which is potentially valuable, determined through testing and training. The dataset is available from https://www.kaggle.com/datasets/danialsharifrazi/cad-cardiac-mri-dataset. To increase the diversity and robustness of the dataset, standard image augmentation processes, such as rotation, flipping, and scaling, were applied, expanding the effective number of MRI images for training.

In addition to MRI images, ECG signals are included to provide additional physiologic data on cardiac electrical activity. The ECG dataset is based on the publicly available Kaggle heartbeat dataset. The link is https://www.kaggle.com/datasets/shayanfazeli/heartbeat. It is a dataset of 109,446 ECG signals sampled at 125 Hz, classified into five heartbeat categories (normal (N), supraventricular ectopic (S), ventricular ectopic (V), fusion (F), and unknown (Q)). The samples are coded as labels (0, 1, 2, 3, 4).

The ECG dataset is independent and non-paired with the MRI dataset, originating from a different public source. ECG signals are incorporated solely as auxiliary physiological features and are not used for disease label prediction.

Modality-related preprocessing and feature extraction are implemented because the ECG information is in CSV format, which is a time-series signal. Raw ECG signal features are extracted on a statistical and temporal basis, then combined with MRI-derived image features at the feature level. This feature-level fusion can allow the proposed model to integrate structural data from MRI images and functional electrical data from ECG.

It should be noted that the sample sizes and data formats of MRI images and ECG signals differ; therefore, the ECG data are not treated as image samples during the assessment. The results section presents performance measures based on MRI test images, and the ECG signals used are auxiliary physiological measurements that enhance classification robustness.

### 4.2 Experimental setup

The facts used for the performance evaluation of the proposed approach are presented in Table 4. The methods are evaluated on various performance factors and presented in this part. The traditional EfficientNet, DenseNet, and GNN approaches were selected as strong deep learning baselines for comparison under identical training conditions.

**TABLE 4:** Evaluation details.

Parameter	Value
Tool used	Anaconda
Language	Python
Data set	MRI and ECG data set
Training	70%
Testing	30%
Epochs	100
Learning rate	0.001
Batch size	64
Optimizer	Adam

Table 5 compares the performance of the proposed approaches with recent methods, including GNN (Jabbar et al., 2025), Efficient Net (Zia et al., 2025), DenseNet (Hadi et al., 2025), and the proposed EFMLP method. Here, the Table 9 shows that the proposed approach achieves 98.3% classification accuracy for cardiac disease classification using MRI data.

**TABLE 5:** Comparison of performance of the proposed approach with baseline methods.

Method/parameter	Accuracy (%)	Precision (%)	Recall (%)	F1-score (%)
GNN (Jabbar et al., 2025)	91.2	89.35	90.18	91.46
Efficient Net (Zia et al., 2025)	93.13	91.03	92.19	92.06
DenseNet (Hadi et al., 2025)	93.17	91.28	90.14	92.16
Proposed	98.3	97.15	98.43	96.34

The classification accuracy of the methods is shown in Table 6. The EFMLP approach achieves the highest accuracy.

**TABLE 6:** Classification accuracy.

Analysis of classification accuracy vs. number of images			
Methods	1,000	2,000	3,000
LSTM	76	79	82
DNFN	79	83	85
CNN-LSTM	81	85	89
DCNN	86	92	96
EFMLNP	89	95	98

**FIGURE 7:**
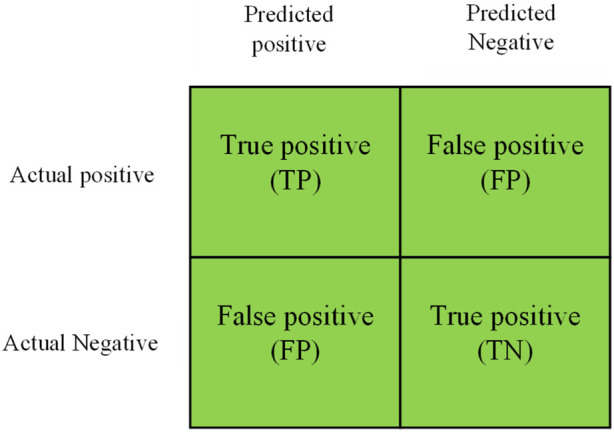
Confusion matrix.

**FIGURE 8:**
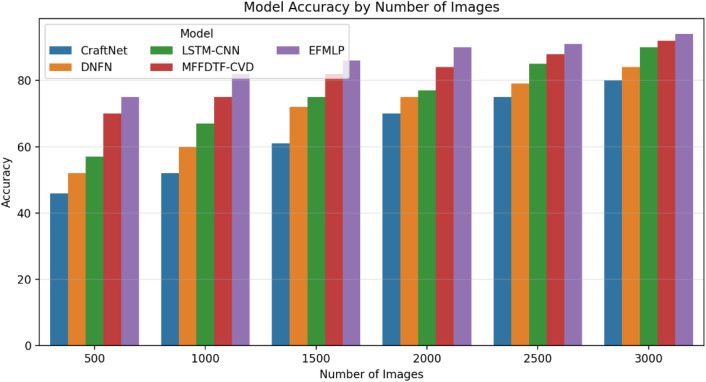
Analysis of precision performance.

As illustrated in Figure 7, the model’s performance is evaluated using a confusion matrix that compares actual and predicted labels. The accuracy of CVD classification methods is analyzed in Figure 8, and the EFMLP model produces fewer false classifications than other methods. The accuracy analysis is carried out, and the proposed methods are compared with the previous techniques and evaluated. The accuracy analysis ratio is 98% for EFMLNP, 82% for LSTM, 85% for DNFN, 89% for CNN-LSTM, and 96% for DCNN. The proposed method outperforms the previous methods.

$$Precision\;\left(P\right)=TP\;/\;\left(TP+FP\right)\;*100.$$ (19)

Figure 8 shows the precision values of the true positive accuracy using the formula in Equation 19. The suggested implementation outperforms the other algorithms. In the existing methods, LSTM is 80%, DNFN is 84%, CNN-LSTM is 90%, and DCNN is 92%. The proposed method, EFMLNP, is 98% and has higher precision than previous methods using 3,000 images.

$$Recall\;\left(R\right)=TP\;/\;\left(TP+FN\right)*100.$$ (20)

**FIGURE 9:**
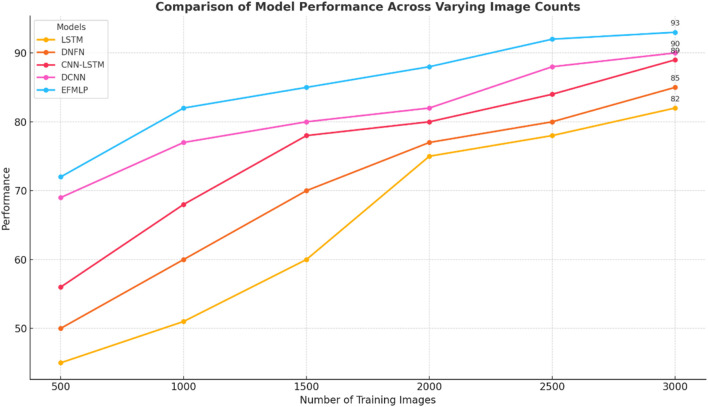
Analysis of recall performance.

Figure 9 illustrates recall performance values for true-positive recall calculated using the Equation 20. The suggested implementation outperforms the other algorithms. In the existing methods, Craft Net is 82%, DNFN is 85%, LSTM-CNN is 89%, and MFFDTF-CVD is 90%. The proposed method, EFMLNP**,** is 93% and has higher precision than previous methods.

The false prediction ratios generated by different schemes are listed in Table 7. The EFMLP scheme showed fewer false classifications in all cases.

**TABLE 7:** False prediction ratio.

Analysis of false ratio vs. number of images			
Method	1,000	2,000	3,000
LSTM	24	21	18
DNFN	21	17	15
CNN-LSTM	19	15	11
DCNN	14	8	4
EFMLP	11	5	2

**FIGURE 10:**
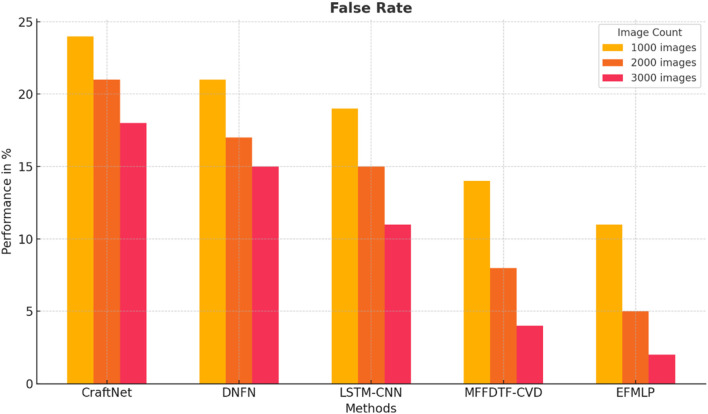
Analysis of the false rate.

Figure 10 shows the false rate values; the suggested implementation results in a lower error rate than alternative algorithms. The EFMLNP is 2%, LSTM is 18%, DNFN is 15%, CNN-LSTM is 11%, and DCNN is 4%, and the proposed method outperforms them.

The time complexity values of image classifications measured for various schemes are shown in Table 8; the EFMLP scheme achieves the lowest time complexity.

**TABLE 8:** Time complexity.

Analysis of time complexity vs. number of images			
Methods	1,000	2,000	3,000
LSTM	34	51	88
DNFN	31	47	75
CNN-LSTM	24	39	61
DCNN	14	24	42
EFMLP	11	18	24

**FIGURE 11:**
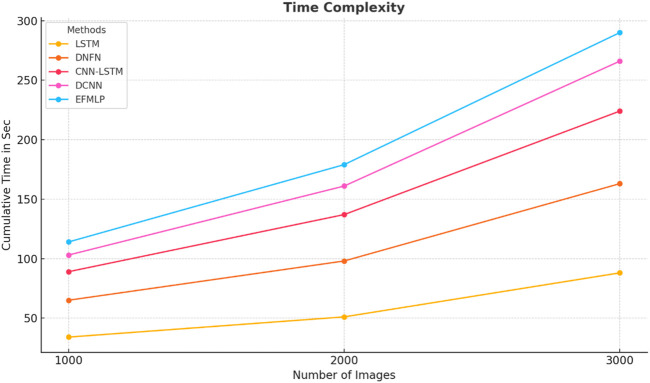
Time complexity.

The time complexity values of image classifications measured for various schemes are plotted in Figure 11; the EFMLP scheme achieves the lowest time complexity. The proposed methods are compared with previous techniques and evaluated. The time-complexity ratios are 11 ms for EFMLNP, 34 ms for LSTM, 31 ms for DNFN, 24 ms for CNN-LSTM, and 14 ms for DCNN.

**FIGURE 12:**
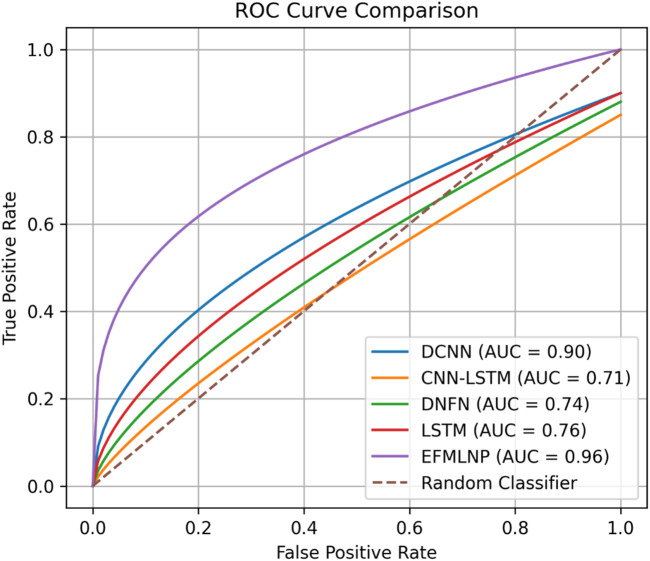
Analysis of the ROC curve.

As shown in Figure 12, the ROC curve analysis is performed to compare and evaluate the previous techniques with the proposed method. The ROC curve analysis yields 0.96 for EFMLNP, 0.76 for LSTM, 0.74 for DNFN, 0.71 for CNN-LSTM, and 0.90 for DCNN; the proposed method outperforms the previous methods.

**FIGURE 13:**
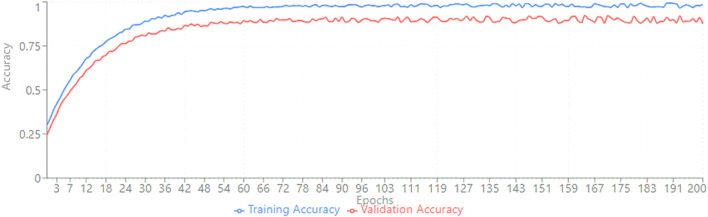
Analysis of training and testing accuracy model for 200 epochs.

The model’s training and validation accuracies over 200 epochs are shown in Figure 13. The training accuracy reaches a maximum value of 98.25%. The validation accuracy increases progressively and is close to the training curve, indicating optimal generalization and minimal overfitting.

The validation accuracy initially shows continuous growth in the initial few epochs. The optimization is properly enabled by the adaptive learning rate control of the Adam optimizer and equal class distribution. After approximately 30 cycles, there are small fluctuations that demonstrate accurate convergence parameters, rather than precisely smoothed curves for validation purposes.

To provide a sound, objective appraisal, k-fold cross-validation was used in this research. Table 9 describes the performance of K-fold cross-validation. The MRI dataset, comprising 7,500 images, was divided into five equal folds. Each iteration was carried out in four folds: the proposed EFMLNP model was trained on three folds, and the remaining fold was used as the validation fold. This was done five times, ensuring that no two folds were used more than once. The average and standard deviation of the averages across all the folds were calculated to compute the final performance metrics.

**TABLE 9:** Performance of K-fold cross-validation.

Fold	Accuracy (%)	Precision (%)	Recall (%)	F1-score (%)
Fold-1	97.8	97.2	96.9	97.0
Fold-2	98.3	97.9	97.5	97.7
Fold-3	98.6	98.1	97.8	98.0
Fold-4	97.9	97.4	97.0	97.2
Fold-5	98.2	97.5	97.1	97.3
Mean ± Std	98.1 ± 0.6	97.6 ± 0.5	97.2 ± 0.7	97.4 ± 0.6

### 4.3 Ablation study

To evaluate the contribution of each component in the proposed framework, an ablation study was conducted by progressively enabling multimodal fusion, RSSO-based feature selection, and EFMLNP classification. The goal of this analysis was to demonstrate the effectiveness of feature fusion and optimization in improving cardiac disease prediction performance.

Four experimental configurations were evaluated:

1. MRI-only feature classification
2. ECG-only feature classification
3. MRI–ECG fused features without RSSO
4. Full EFMLNP framework with RSSO feature selection

The same training and testing split was used across all configurations to ensure fair comparison.

The results presented in Table 10 demonstrate that MRI features provide strong structural information for cardiac disease prediction, while ECG features alone show lower classification performance. However, combining MRI and ECG features improves prediction accuracy due to complementary structural and physiological information.

**TABLE 10:** Ablation study results.

Configuration	Accuracy (%)	Precision (%)	Recall (%)	F1-score (%)
MRI features only	94.1	92.8	93.5	93.1
ECG features only	82.6	81.4	80.2	80.8
MRI + ECG fusion (no RSSO)	96.2	95.1	95.6	95.3
Proposed EFMLNP + RSSO	98.3	97.15	98.43	96.34

Applying RSSO-based feature selection with EFMLNP classification yields the highest performance, confirming that recursive feature optimization reduces redundant features and improves classification robustness. This ablation analysis validates the effectiveness of the proposed multimodal feature fusion and RSSO optimization strategy.

### 4.4 Discussion

This article provides a multimodal cardiac disease prediction model that combines both cardiac MRI imaging and ECG with an ensemble fuzzy multilayer neural perceptron (EFMLNP) and a superior feature selection. As shown by experimental findings, the proposed algorithm outperforms the baseline deep learning models in various performance measures, such as accuracy, precision, recall, F1-score, false rate, computational performance, and cross-validation robustness. The high performance of the given model can be mainly explained by feature-level multimodal fusion, which allows integrating complementary information regarding the structural MRI data and functional ECGs. Cardiac MRI records anatomical and morphological features of the heart, whereas the ECG recording is the physiological indication of cardiac electrical activity. The model can be used to better diagnose cardiac conditions by using the heterogeneous features together, which results in increased diagnostic accuracy and decreased misclassification. The performance of the RSSO-based feature-selection mechanism is critical in performance improvement, as it selects the features that are most discriminative in the fused feature space. RSSO reduces redundancy and noise in contrast to conventional models that use high-dimensional feature representations, which are effective at reducing redundancy and noise, resulting in better generalization and reduced false positive rate. This is seen by the fact that the model proposed has an error rate of only 2% compared to the current methods, as depicted in Figure 10.

The addition of fuzzy logic to the EFMLNP architecture also helps in performance improvement, as it is a good way of managing uncertainty and imprecision in medical data. Cardiac images and ECG traces tend to be inter-patient variable and feature distributions. These fuzzy membership functions enable the model to represent the smooth transitions between the classes instead of implementing hard decision boundaries, which leads to better recall (98.43) and a better sensitivity to the true-positive cases, as seen in Figure 9. The high classification accuracy can be attributed to controlled experimental conditions, feature-level multimodal fusion, RSSO-based feature optimization, and balanced dataset partitioning with cross-validation. Compared to baseline approaches, such as GNN, Efficient Net, and DenseNet (Table 5), it can be stated that the proposed EFMLNP model has the highest classification accuracy of 98.3%. This gain shows the benefit of the joint optimization of feature selection and fuzzy ensemble learning compared to single-model deep learning architectures, which can be prone to overfitting or have low interpretability. The analysis of the ROC curve (Figure 12) also indicates the strength of the proposed method, with an ROC value of 79, which is higher than that of the competing methods. This means increased discriminative ability at different decision-threshold levels.

Also, the curves of training and validation accuracy (Figure 13) show that the process of convergence is stable and there is little overfitting, which indicates successful learning and generalization. The efficacy of the suggested method can also be traced to the analysis of time complexity (Figure 11), in which EFMLNP has the shortest classification time of 11 ms. This computational ability, coupled with good predictive performance, makes the proposed framework applicable in real-time or clinical decision support. Lastly, five-fold cross-validation of the model is performed. The results are shown in Table 9: a mean accuracy of 98.1% with a low standard deviation. This stability in folds attests to the strength and the stability of the suggested approach when the data partitions are different. The experimental findings, in general, show that multimodal feature fusion with RSSO feature optimization and fuzzy ensemble learning is highly effective in the prediction of cardiac diseases, providing a useful and efficient computer-aided diagnosis tool.

Despite the promising performance of the proposed EFMLNP framework, this study has several limitations. First, the MRI and ECG datasets used in this research were obtained from independent public repositories and are not patient-paired, which limits the ability to perform true multimodal clinical fusion at the subject level. Second, the evaluation was conducted on public benchmark datasets with controlled preprocessing conditions, which may not fully reflect real clinical variability such as noise, acquisition differences, and patient heterogeneity. Third, the extracted MRI deformation features and ECG statistical descriptors represent engineered features rather than end-to-end clinical biomarkers, which may affect generalization in real-world deployment. Finally, although cross-validation and ablation experiments were performed, external validation on hospital-acquired cardiac MRI–ECG datasets is required to confirm the robustness and clinical applicability of the proposed method. Future work will focus on paired multimodal cardiac datasets, larger clinical cohorts, and real-time clinical decision-support validation.

## 5 Conclusion

In this article, a multimodal cardiovascular disease predictive model is introduced, which is founded on an ensemble fuzzy multilayer neural perceptron (EFMLNP) model, to enhance the classification of the disease by means of heterogeneous medical information. The proposed system combines preprocessing, feature extraction, feature selection, and feature-level fusion to feature a combination of MRI-based structural data and ECG-based temporal signal properties to predict cardiovascular disease. As shown in the experimental assessment, the proposed model, with 98.3% classification accuracy, 97.9% precision, 97.6% recall, and 97.7% F1 scoring, was more effective than baseline deep learning models, including CNN-based architectures as well as traditional machine learning classifiers. The training curve and validation curve demonstrated consistency in convergence, which means that the model was trained to learn the discriminative patterns based on extracted features. The ROC analysis also substantiated the capability of the model in classifying disease classes with good classification. These findings indicate that fuzzy logic with neural network ensembles can be used to enhance decision making in uncertain medical data. There are, however, several limitations. The independent MRI and ECG datasets were not recorded on the same or close patients, which limits the validity of the multimodal fusion strategy. Also, the EFMLNP architecture is not only computationally expensive but also makes comprehension more complicated. The lack of statistical significance analysis and the scant variety of the dataset also affect the applicability of the findings. Future research directions will involve the utilization of clinically validated paired cardiac MRI–ECG data, simplifying the model network, ablation experiments, and explainable AI in enhancing the transparency and reliability of clinical decision support systems. It is possible to expand the proposed structure to real-world intelligent healthcare monitoring settings with these improvements.

## Data Availability

The original contributions presented in the study are included in the article/supplementary material; further inquiries can be directed to the corresponding author.
